# Urticaria

**Published:** 1890-12-27

**Authors:** 


### URTICARIA.

The symptoms of urticaria, or "nettle rash," are so well defined that a mistake in diagnosis can rarely occur. White prominences, taking the form of round tubercles or white stripes, or wheals of varying length, shown up on a scarlet or crimson ground are distinctive. So also, in a secondary degree, is the accompanying burning, pricking, itchings, or stinging. But the sudden manner in which the eruption occurs is often the cause of great anxiety on the part of the patient. Although an ordinary case of urticaria is so easily diagnosed by the professional eye, the malady still present, in various forms, which may not be quite so readily differentiated. The diversity of symptoms have suggested a variety of appellations. Thus there is *urticaria evanida* or evanescent; u. *persistans*; u. *conferta*; u. *subcutanea*; u. *tuberosa*. In addition, there is "factitious urticaria." In an article on urticaria, lately published by Professor Otto Kahler, of the University of Vienna, it is stated that all the forms of urticaria may be further classified into those which run a rapid course, and others that drag on through months, or even years. And Mr. Hutchinson latterly observed in the *Archives of Surgery*, that "the cases of urticaria, which terminate rapidly, are generally principally characterised by wheals." Mr. Hutchinson also states that "there is a peculiar form of erythematous urticaria, and in such cases contrary to what is usual in urticaria, the patients are most comfortable when warm in bed." Another example of a mixed form is *eczema-urticaria*, or urticaria occurring in conjunction with eczema. Closely allied to, if not indeed to be regarded as a variety of urticaria, is so called *lichen urticatus*, which is marked by the efflorescence of thickly disseminated little papules, the seat of intense prurigo. Although urticaria, being a symptom itself, has no special constitutional symptoms, it may be associated with general derangement of the system, which has led to the term "febrile urticaria." But as the late Erasmus Wilson well observed, "such a condition ought rather to be designated *urticaria cum febre*." The association of urticaria with digestive derangements has also led to the term *urticaria ab ingestis*.

Dr. Henry Waldo, of Aberdeen, recently read before the Bristol Medico-Chirurgical Society a case of "Factitious Urticaria." The patient was a male, æt. 29, who suffered much from acute dyspepsia. There was such excessive irritability of the cutaneous nerves that wheals could be excited by local irritation. If letters were inscribed with any bluntly-pointed instrument, white letters with pink borders stood out in bold relief on the skin. Dr. Waldo remarks that the etiology of this affection is much the same as the etiology of ordinary urticaria, and it may be produced by insect bites, or by any kind of external visitation. Dr. A. J. Harrison, physician to the Bristol General Hospital, also brought before the same society two cases of factitious urticaria. The first was that of a young man, on whom the slightest blow would be followed by a distinct whealing. He had no marked indigestion nor any other ailment. The other case was the brother of the first, who suffered much in the same manner but in addition he had been liable for several years to attacks of epistaxis. In both patients wheals were more easily produced in hot than in cold weather. Dr. Harrison attempts an explanation thus: "First, the marking of the skin sets up an irritation which is conveyed to the capillaries of the part through the vaso-motor system; the capillaries dilate, causing the red lines to appear; then the adjoining surfaces participate in the action, and the suffusion spreads from the parts first irritated. Shortly, the capillaries first affected begin to get paralysed, white distinct wheals appear, and serum is forced out. By-and-by the capillaries begin to contract again, the suffusion gradually disappears, and the serum of the wheals is re-absorbed." This may be correct as regards the mechanism of the affection, but it leaves us quite in the dark as to the reason why the affection occurs.

Dr. Harrison further observes that cases of factitious urticaria should be looked at from a medico-legal point of view. Suppose a school teacher had given the younger boy a few smart strokes with a cane. The appearance would be quite out of proportion to the severity of the punishment, and a very erroneous opinion might be readily formed, and a charge of undue violence be very easily set up.

Most dermatologists now regard urticaria as a neurotic disorder, the functions of the nerves being so intimately intermingled with the symptoms of urticaria. We know that urticaria is often excited by substances taken into the stomach, especially by shell fish. It may also be excited by certain drugs as copaibæ, cubebs, or quinine. The view of the neurotic nature of the affection is further strengthened by the eruption being sometimes accompanied by spasmodic cough, sneezing, or asthma. Uterine irritation is another source of urticaria. Dr. Waldo, of Aberdeen, mentions the case of a lady in whom the advent of strangers caused urticaria, and this sensitiveness so much increased, that at last a knock or a ring at the door would determine an immediate attack. There are persons who cannot even see crabs without suffering from the rash. In such cases the affection is plainly a nervous one. Some hysterical females are very susceptible to factitious urticaria. Everything in urticaria points to its being primarily a vaso-motor disturbance, direct or reflex, central or peripheral. Unna thinks the wheal is produced by a spasm of the large veins of the skin, which normally serve to carry off the lymph.

But notwithstanding the admission of urticaria into the class of neuroses it does not follow, as some have argued, that the best treatment is always by drugs adapted to the neurotic condition. If urticaria can be clearly traced to articles of food, such articles should be got rid of by vomiting if they still remain in the stomach, otherwise by aperients. Urticaria is sometimes attended by sickness and attempts to vomit, and the proper way to relieve the urticaria is to assist nature. Urticaria is also sometimes attended by colicky pains and purging, which latter may also be assisted. Again, the question may be investigated, Is there a gouty diathesis? If so, this condition should be treated. Urticaria may occur in jaundice; it is not unusual in albuminuria and glycosuria; it may be associated with rheumatism, purpura, and intermittent fever. Sometimes it occurs during acute nephritis. It is, therefore, manifest that special treatment by remedies directed against the urticaria would not be desirable. There are, however, cases of urticaria which do not appear associated with other ailments, and such cases usually happen in neurotic constitutions. In a recent number of the *Journel de Medicin* we observe that Mons. Nitot advises antipyrin for chronic urticaria of this description, which he believes to be essentially of nervous origin. A case is related of relief thus afforded to a patient who had been treated without benefit by purgatives, alkalies, quinine, and other medicines. Eight grams of antipyrin were given daily. After the first dose the urticaria was less, and at the end of a week it was completely gone. We cannot, however, expect such success in all cases. Numerous drugs have apparently been beneficial and afterwards failed. The list recommended is a long one, comprising salicylates, quinine, lime juice, sulphonal, strophanthus, canabis indica, belladonna, arsenic, bromides, nux vomica, etc. Several practitioners have also extolled ergot. We think Mr. Harrison, of Bristol, has well summed up when he says (*British Medical Chirurgical Journal* of March last): "At the best drugs seem to be rather inoperative." Similarly with local applications, what seems to agree with and to relieve some persons often rather aggravates suffering in others. In our experience a 2 p.c. solution of carbolic acid has been generally the most useful. A lotion of lime water inspissated with oxide of zinc, one part of the latter to eight of the former, was recommended by Erasmus Wilson.

In conclusion, we may observe that recurrent urticaria in bilious subjects has frequently been at least temporarily cured by a course of Carlsbad water. But the urticaria is liable to return if the patient recurs to free living after the Carlsbad course.

